# Analytical Protein Microarrays: Advancements Towards Clinical Applications

**DOI:** 10.3390/s17020256

**Published:** 2017-01-29

**Authors:** Ursula Sauer

**Affiliations:** AIT Austrian Institute of Technology GmbH, Center for Health and Bioresources, 3430 Tulln, Austria; ursula.sauer@ait.ac.at; Tel.: +43-50550-3527

**Keywords:** point-of-care, arrayed biosensors, miniaturization, optical signal transduction, protein biomarker

## Abstract

Protein microarrays represent a powerful technology with the potential to serve as tools for the detection of a broad range of analytes in numerous applications such as diagnostics, drug development, food safety, and environmental monitoring. Key features of analytical protein microarrays include high throughput and relatively low costs due to minimal reagent consumption, multiplexing, fast kinetics and hence measurements, and the possibility of functional integration. So far, especially fundamental studies in molecular and cell biology have been conducted using protein microarrays, while the potential for clinical, notably point-of-care applications is not yet fully utilized. The question arises what features have to be implemented and what improvements have to be made in order to fully exploit the technology. In the past we have identified various obstacles that have to be overcome in order to promote protein microarray technology in the diagnostic field. Issues that need significant improvement to make the technology more attractive for the diagnostic market are for instance: too low sensitivity and deficiency in reproducibility, inadequate analysis time, lack of high-quality antibodies and validated reagents, lack of automation and portable instruments, and cost of instruments necessary for chip production and read-out. The scope of the paper at hand is to review approaches to solve these problems.

## 1. Introduction

In a review article from 2012, Zhu and Qian from John Hopkins University School of Medicine claim, “As a powerful technology platform, it would not be surprising if protein microarrays will become one of the leading technologies in proteomic and diagnostic fields in the next decade” [1]. Already in the past protein microarrays have been hyped as future key technologies in a wide field of applications, from basic research to industrial applications [2]. In some respects the promises materialized, as for instance protein microarrays are now widely used in basic research and drug screening. On the other hand microarrays are still far from being used routinely in clinical practice and point-of care. The question arises what features have to be implemented and what improvements have to be made in order to fully exploit the technology. In the past we have identified various obstacles that have to be overcome in order to promote protein microarray technology in the diagnostic field. The main challenges encountered are the following:

(i) Low reproducibility: The accurate quantification of a signal following a binding event on the chip relies on low intra- and inter- spot variability in the first line and results from a highly controlled probe dispensing. Optimization of the experimental set-up including quality of chip substrate and coating technique, print buffers, immobilization strategy, dispensing (pins), probe concentration, blocking and assay design, as well as image and data analysis is crucial in order to decrease variability [3,4,5].

(ii) Low sensitivity: For proteins there is no efficient signal amplification method established such as Polymerase Chain Reaction (PCR) for DNA microarrays. This makes the detection of low abundant proteins especially difficult and sensitive methods a must. Crucial factors for high sensitivity are the intrinsic affinity of the biorecognition element (BRE) as well as its immobilization rendering biological activity and accessibility. Further high quantum yield labels and efficient detection techniques promote low detection limits [6,7].

(iii) Quality of antibodies: antibodies are the centerpiece of immunoassays; their specificity and constant quality are a determining factor for the success of on-chip assays. Batch-to-batch variability, crossreactivity, low activity and low affinity of commercial antibodies may pose major problems for the reliability and reproducibility of clinical tests [8,9,10].

(iv) Time to result: Quick diagnosis and immediate treatment is what a clinician expects from a bioanalytical method [11]. Not only reaction kinetics is a parameter for the time a test takes. Assay design in terms of incubation- and washing steps necessary, read out and even more data analysis can significantly increase the time a test takes from sample input to answer output.

(v) Miniaturization and system integration: Standard microarray instruments are big and heavy, but also most of the competing techniques are not portable, e.g., chromatographic techniques such as high performance liquid chromatography (HPLC), mass spectrometry (MS), and flow cytometry, depend on big instruments in central labs. Making instruments for microarrays portable will be a major driver for market entry [12].

(vi) Production cost: Even the best diagnostic tool will only succeed in the health market if it is cheap enough to be accepted by health insurances for reimbursement. The cost factor has to be considered when designing the assay, choosing the biorecognition elements, substrates, labels and surface chemistries. Integration of nanotechnology for signal enhancement for instance may increase production cost tremendously, while miniaturization can reduce reagent and sample consumption.

## 2. General Background

The concept of immunoanalytical microarray technology emerged in the late 1980s. With the first reports of Ekins and co-workers fluorescence immunoassays started to replace methods using radioisotopic labels dominating medicine and other biologically-related fields at that time. But the authors not only aimed at a non-isotopic immunoassay method, they also suggested the concept of “microspot immunoassays” on solid supports allowing multianalyte measurements [13,14]. The general concept of a protein microarray comprises arraying of capture probes to discrete positions onto a solid support, sample incubation, and optical detection of the analyte binding. In some respects protein microarrays outperform conventional chromatographic techniques such as GC-MS or HPLC-MS. Key features are high throughput and low cost due to minimal reagent consumption, multiplexing, fast kinetics and hence measurements, and the possibility of functional integration. Miniaturization is one of the pre-requisites for the latter one. Nowadays microarray technology is embracing nanotechnology as well; as for instance by integration of functional elements in the nanoscale serving as labels, separation and support material for biorecognition elements, and for signal enhancement [15,16,17].

Depending on the application Zhu and Snyder [18] define two types of protein microarrays: analytical and functional [1]. Functional protein microarrays are developed for the study and elucidation of the function and interaction of various biological molecules, while analytical protein microarrays aim at the quantitative detection of analytes in various samples. Many fundamental studies have been conducted in a large variety of molecular and cell biology areas, using protein microarrays. Functional protein microarrays have been developed for instance for analysis of enzyme kinetics [19], analysis of expression profiles [20,21], and understanding disease at molecular level [22]. Analytical protein microarrays are often implemented using antibodies as recognition and detection elements and are thus referred to as “on-chip immunoassays” herein, albeit antibodies may be replaced by artificial affinity molecules. On-chip immunoassays have been shown to detect a vast range of analytes in numerous applications, such as clinical diagnosis [23] and patient stratification [24], drug development [25], environmental monitoring [26], and food safety [27].

**Figure 1:**
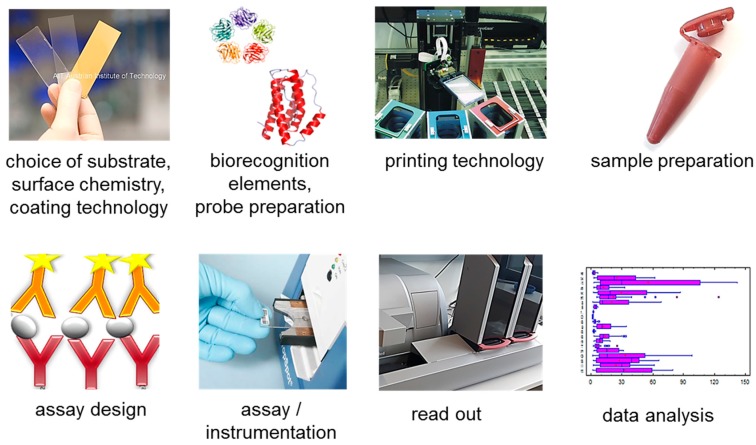
Components of protein microarray production and processing. Work flow from left to right: Preparation of the immobilization surface, probe preparation, printing, sample preparation, assay design, instrumentation, read-out and data analysis.

Each spot in a microarray can be seen as a reaction chamber for a biosensor. A biosensor is defined as analytical device incorporating a biological or biologically derived sensitive ‘recognition’ element and a transducer that converts a biological response into a digital electronic signal [28]. Microarrays (or the equivalent term “biochips”) are sometimes referred to as arrayed biosensors. In contrast to a classical biosensor microarrays are often not regenerable (with a few exceptions e.g., [29]), notably in a medical application a disposable chip is preferred, and online measurements (in situ monitoring, real-time measurements) are still rare and depend on the integration of microfluidics. After molecular recognition of the target molecule microarrays need a) an additional detection step (e.g., adding labeled detection antibodies) and often b) a separation step (washing off unbound material. Figure 1 depicts key components of a protein chip experiment, which will be discussed in the following.

In microarray technology multiplexing is achieved by positional encoding. Each position in the array (i.e., each spot) represents a specific capture probe and hence a single label (“color”) is usually sufficient for read out of hundreds of different analytes. To overcome some problems with slow diffusion and low sensitivity of big arrays on solid supports, so called “solution arrays” were introduced. Here encoding is accomplished either by fluorescently labelled microbeads or by barcoded particles [30]. But this approach has the disadvantage of a limited number of analytes.

## 3. Supports and Immobilization Strategies

**Figure 2:**
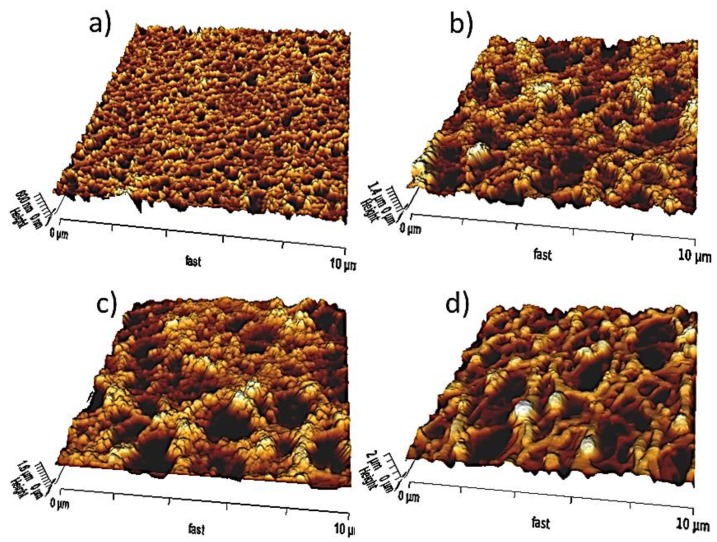
AFM scans of four commercial nitrocellulose slides for protein adsorption. (**a**) UniSart 3D (Sartorius, Göttingen, Germany) (**b**) Oncyte Nova (Grace Biolabs, Bend, OR, USA) (**c**) FAST slide (Maine Manufacturing, Portland, ME, USA) (**d**) Super Nitro (ArrayIt, Sunnyvale, CA, USA). Root Mean Square Roughness increases from (**a**) 62 nm to (**d**) 274 nm.

Microarrays require surfaces to be biocompatible and rich in binding sites. The choice of the surface chemistry is governed by some technology inherent requirements such as low background (for fluorescence read out that means low autofluorescence at the excitation wavelength), low unspecific binding (often referred to as antifouling properties); providing good stability, and accessibility of the probes. Last but not least, it depends on the solid support which is often glass, plastic, silicon dioxide or gold. Hydrogels [31,32], gel pads [33], and particularly nitrocellulose [34] are popular 3D substrates for protein microarrays. In Figure 2. Atomic Force Microscopy (AFM) images of four commercial nitrocellulose slides for protein adsorption with varying thickness, roughness and pore size are depicted, autofluorescence and binding site density for instance have to be considered before use. BREs can also be entrapped in transparent porous sol-gels, protecting and stabilizing them, given that the sol-gel preparation is modified to be biocompatible (i.e., reduced alcohol content and introduction of appropriate buffers). Sol–gel immobilized biomolecules are reported to retain their structural integrity and biological activity [35].

Most commonly planar surfaces are used providing reactive aldehyde-, epoxy-, isothiocyanate, amino-, or mercapto-groups. N-Hydroxysuccinimide (NHS) esters and aldehyde groups are amine reactive, maleiimide reacts with thiol groups, epoxides bind both, amine and thiol, and surfaces with exposed amino groups may bind to EDC/NHS activated carboxy groups. Functional groups of amino acids protruding from the protein surface such as lysines and cysteines can be employed for direct covalent attachment. Some latest developments and controversial views on immobilization are highlighted in the following.

### 3.1. Pros and Cons of Oriented Antibody Immobilization

In contrast to randomly immobilized antibodies (e.g., via adsorption) and statistically oriented antibodies (i.e., covalent binding to certain accessible amino acids), where preferred binding of functional moieties to a surface is assumed, oriented antibody immobilization aims at optimal presentation of the Fab regions for most efficient antigen capturing. Pros and cons of orientation of the capture molecules have been discussed for a long time. While for “micro”arrays orientation of antibodies may not have such a big impact, for “nano” spots high binding densities of oriented capture molecules may become crucial in order to generate a signal [12]. Clearly, orientation is also very important for methods where distance is a crucial parameter such as Förster resonance energy transfer (FRET). Orientation may go along with chemical modification of antibodies and the question whether the immobilization strategy ends up with a higher number of biologically active antibodies often remains open. Protein A and Protein G, naturally occurring proteins from *Staphylococcus aureus* and *Streptococcus sp.*, exhibit a strong affinity to the Fc part of IgGs of some species as for instance of human IgG, Rat IgG2c and Mouse IgG2a (for Protein A). In principle this end-on attachment allows control of antibody orientation presenting the Fab regions toward the sample solution and does not require modification of the antibodies. The dissociation of the antibodies at low pH (such as 0.1 M glycine.HCl pH 2–3) on the other hand can be used for regeneration of chips. Covalent binding of antibodies using amine reactive surfaces will result in statistical orientation, but an intermediate layer of protein G is not oriented either when using amine coupling and hence first and foremost orientation of the protein G itself should be pursued in order to really control antibody orientation [36]. This may be obtained with thiolated protein G self-assembling on gold [37] as this a three-fold more efficient antibody immobilization compared to covalent binding via NHS ester was obtained, which may also be attributed to low efficiency of the esters formed.

**Figure 3:**
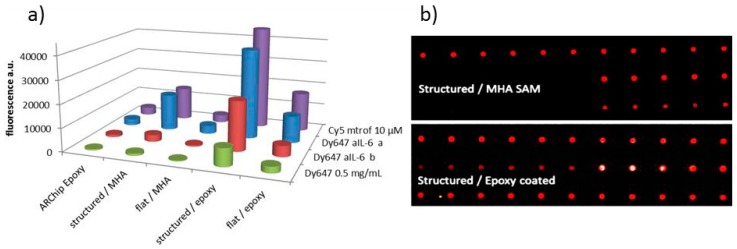
(**a**) Comparison of the fluorescence signals originating from spotted biomolecules labelled with fluorescent dyes on ARChip Epoxy (glass) and flat and structured gold substrates coated with mercaptohexadecanoic acid/carbodiimide (MHA/EDC) or epoxy resin Epikote 157; (**b**) Example of microarray image scan comparing the two covalent binding chemistries on a nanostructured gold chip. Ursula Sauer, unpublished results.

In [38] the authors compare covalent binding of antibodies to surface plasmon resonance (SPR) gold chips via alkanethiol self assembled monolayers (SAMs) to protein G mediated binding and to an oriented calixarene-based immobilization (ProLinker™). The protein is captured by the calix crown derivative by a host-guest interaction of the ionized amine groups and the crown moieties [39]. The ProLinker strategy showed significantly lower LOD and wider measurement range compared to the two other immobilization strategies and worked also in pure urine and diluted serum. The covalent attachment employing an alkanethiol SAM in combination with carbodiimide/*N*-hydroxysuccinimide (EDC/NHS) chemistry yielded very low amount of antibody on the surface, the authors assume a very low density of NHS-esters formed [38]. This is in accordance with our own unpublished results. A thin layer of epoxy resin coated onto gold outperformed conventional alkanethiol chemistry (Figure 3) modified with EDC on flat and nanostructured gold substrates for covalent binding of labelled biomolecules. Another approach for oriented immobilization on gold using recombinant bispecific antibodies is reported in Watanabe et al. [40]. An antigold antibody was combined with anti-lysozyme antibody via a rigid linker. The lysozyme binding capacity of this immobilized construct was calculated as 82% from the amounts of immobilized antibody and antigen compared to 59% using conventional EDC/NHS chemistry.

### 3.2. Surface Chemistries for Small Molecules

The detection of small molecules is particularly challenging. The size of the molecules hampers sandwich immunoassays, only binding inhibition or competitive assays are possible. As a consequence immobilization of target molecules is necessary in a way providing good accessibility for the detection antibodies. This task has been accomplished by coating conjugates of small molecules with big proteins, e.g., bovine serum albumin (BSA), human serum albumin (has), horseradish peroxidase (HRP), keyhole limpet hemocyanin (KLH) etc. [41,42]. Another way to present molecules on a surface is to either coat them onto microparticles which are then arrayed onto the chip [7], entrap them in sol-gels [35] or other porous materials, immobilization onto dendrimers [43,44] or polymer brushes [45,46].

### 3.3. Coating of Substrates

A uniform coating for chip functionalization is a pre-requisite for reproducible and stable binding of biorecognition elements. The choice of a suitable coating technique depends on substrate, chemistry of the functional material and desired film thickness. Dip coating of 1% SU8 (v = 100 mm/min) onto glass for instance yielded about 20 nm film thickness, determined by scanning a scratch in the coating with AFM [47]. Spincoating of the same material, on the other hand, depending on the spin coating parameters resulted in 26 nm (10 s @ 1800 rpm, 30 s @ 300 rpm; acc = 500 rpm/s) and 19 nm layers (40 s @ 4000 rpm, acc = 1300 rpm/s), determined via a TM angular reflectivity spectrum [48]. Using a manual film applicator the wet layer thickness can be chosen as e.g., 15 µm, 30 µm, 60 µm, and 100 µm. The film applicator is used for solutions with high vapour pressure. Metallic coatings can be produced by physical or chemical vapour deposition. Silanization of chips is usually accomplished by liquid phase deposition of the silane in an organic solvent [49,50] or gas phase deposition [51].

## 4. Biological and Biomimetic Recognition Elements (BREs) in Immunoanalytical Microarrays

Biorecognition elements briefly described in the following differ in affinity to the target molecules (Kd values), dynamic range, specificity, size and hence density on a substrate, stability under harsh conditions, shelf life and last but not least production cost. Originally BREs were isolated from living systems such as antibodies, enzymes, receptors, even whole cells may be used. Now a growing number of artificial biorecognition elements are employed for sensing.

### 4.1. Antibodies

Antibodies (immunoglobulins) are glycoprotein receptors of vertebrates serving the immune system for identifying and neutralizing foreign substances. Analytical applications make use of the natural immunoreaction where an antibody is recognizing an analyte with high specificity and sensitivity. The Y shaped antibody molecules feature dimensions of about 14 nm in height, 8.5 nm in width, and 4 nm thickness and are formed by two identical light chains and two identical heavy chains, linked by disulphide bridges and non-covalent bonds. Polyclonal antibodies can only be produced for targets which elicit an immunogenic response on one hand and are not killing the host animal on the other hand. Polyclonal antibodies are produced in a number of mammal species such as mouse, rat, rabbit, goat, donkey, and llama. They are actually a mixture of antibodies produced in different B-cells and target different epitopes. The quality of polyclonal antibodies may differ from batch to batch. Monoclonal antibodies on the other hand are produced by hybridoma technology in only one cell line, as described by Köhler and Milstein [52] in 1975, and hence target only one specific epitope. Cloning and expression of whole antibodies or antibody fragments provides new opportunities. Screening techniques allow isolating mAbs against virtually any target with high affintiy and specificity from large libraries of recombinant antibody fragments [53]. Further, recombinant antibodies can be genetically engineered to self-assemble on biosensor surfaces, or directly modified with markers for detection [54]. For sandwich assays two antibodies specific for the analyte are necessary, often a mix of e.g., a monoclonal capture antibody and a polyclonal labelled detection antibody is used. Instead of a labelled antibody, detection may be accomplished by a third, species specific secondary antibody with a label. Multiplexed quantification of high numbers of analytes such as pesticides, drugs and their metabolites, endocrine-disrupting compounds or other contaminants of food, feed and environment, asks for as many specific antibodies or even antibody pairs. Specificity, cross reactivities, problems with toxic compounds and not to forget very high development costs are limiting the applicability of antibody based systems. A significant number of commercially available antibodies lacked specificity in two studies conducted in 2011 [55,56]. In order to improve the situation Baker [8] suggested sharing information and validation data of commercial antibodies in resources such as: antibodypedia.com; antibodies-online.com; antibodyregistry.org; pabmabs.com/wordpress.

### 4.2. Peptides

Peptides, short polymers of amino acids linked by peptide bonds, have been used as biorecognition elements for proteins, antibodies, DNA, and metallic ions. Peptides with high affinity to targeted analytes, so called mimotopes [57], can be either chosen by screening peptide libraries or are known natural ligands to the target molecule. The production of the specific sequences is accomplished by solid-phase synthesis, and modifications for immobilization and labelling may be included in the process. Peptides are especially useful in combination with environment-sensitive fluorophores, fluorescent resonance energy transfer (FRET) or as part of an excimer, a dimer with a longer emission wavelength than the monomer [58].

### 4.3. Aptamers

Aptamers are artificial nucleic acid ligands which were selected to show high affinity to a certain target. The sequence of the DNA or RNA oligonucleotides is determined by the Systematic Evolution of Ligands by Exponential enrichment (SELEX) process, in which big libraries of artificial oligonucleotides undergo an iterative process of adsorption, recovery and amplification [59]. Still, like for antibodies and MIPs, cross-reactivity of the selected aptamer with structurally similar targets may occur. Producing aptamers in prokaryotic cells sometimes leads to problems with the affinity to targeted proteins from eukaryotic cells, as post-translational modifications may differ and hinder binding of the aptamer [60]. A specific aptamer shows affinity to a peptide, a protein, a cell or a small organic or inorganic molecule comparable to antibodies but with a number of advantages compared to those. First, there is no immunogenic response of an animal needed and hence also very small or toxic targets are possible. Aptamers may be readily manufactured with a linker to bind to a sensor surface and a label for detection without altering the affinity to the target. Further they can be produced and will function under conditions where antibodies fail to work, such as in organic solvents or extreme pH. On the other hand, aptamers and especially RNA aptamers have to be protected against the ubiquitous nucleases. This is accomplished by chemical modification or by using mirror-image nucleotides, so called Spiegelmers^®^ [61].

**Figure 4:**
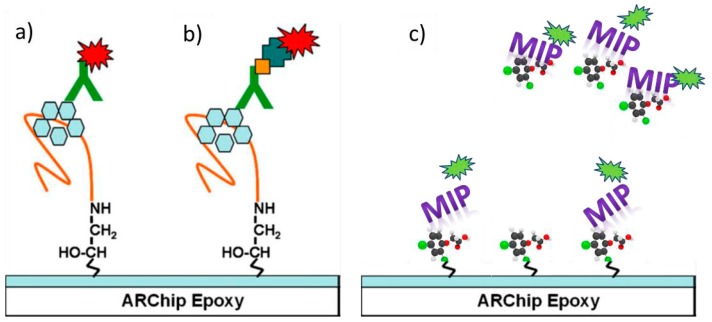
Integration of an aptamer as biorecognition element for a protein in a sandwich immunoassay format with (**a**) a fluorescently labelled antibody or (**b**) a biotinylated antibody and labelled streptavidin. Reproduced from [62] with permission from Elsevier; (**c**) Integration of a fluorescently labelled MIP as detection element in a binding inhibition assay for a small toxin.

Aptamers have been used in various assay formats (direct, competitive, binding inhibition, sandwich assays) alone or in combination with antibodies. In Pultar et al. [62] an aptamer specific to C-reactive protein is used in multiplexed on-chip immunoassays (see Figure 4a,b). The lower affinity of the aptamer shifts the working range of the chip to the desired high serum concentrations of this biomarker for inflammation.

### 4.4. Molecularly Imprinted Polymers (MIPs)

MIPs are cross-linked polymers designed to specifically and selectively interact with target molecules [63]. Monomers displaying functional groups are polymerized together with cross-linkers in the presence of the template molecules. When the template is removed, cavities complementary in size, shape and functionality to the target are created [64]. MIPs are stable and more robust than natural BREs, they work also in extreme environments, such as in the presence of acids or bases, in organic solvents, or at high temperatures and pressures [65]. Limitations of MIPs include cross-reactivities, leaching of template residues, and poor performance in water based solutions [66].

MIPs have been applied for separation and purification mainly; now they are entering the field of drug delivery and detection of molecules, whole cells [67,68] and even viruses [69]. A format for detection of a small toxin via a binding inhibition assay with a labelled MIP is depicted in Figure 4c. In [70] a ready to use epoxy resist was used for hot embossing of lipopolysaccharide and lipoteichoic acid, surface markers specific for Gram-negative and Gram-positive bacteria. The affinity of bacteria imprinted sol-gel films towards their target organisms was found to be governed by the morphology of the cavity and residual surface components entrapped in the imprint surface [68].

## 5. Up-to-Date Patterning of BREs

For protein microarrays there are two standard methods for arraying pre-synthesized capture probes, namely contact and contactless printing. Other techniques such as µ-contact printing (µCP) and nanobiolithography are only of minor commercial importance yet but are gaining more and more interest in the context of miniaturization and system integration. In situ synthesis using photolithography can be applied for DNA directed protein immobilization.

### 5.1. Non-Contact Printing

Inkjet printing is based on the ejection of drops from a nozzle, shot onto a surface. The generation of the drop is accomplished by piezoelectric micropumps, a continuous stream controlled by valves, or thermal inkjet technology, being the first one the most common jetting technique [71]. Non-contact printing can be applied to practically all substrates but is especially apt for damageable surfaces. The piezo voltage has to be optimized for different printing solutions which makes non-contact printing less flexible compared to contact printing. Further, nozzles are very sensitive to damage by contaminations with particles. The probe volume needed to fill the syringes including a dead volume is relatively high compared to the one for a contact printer pin, thus non-contact printing is suitable for printing high number of spots with one printing solution. The high speed a non-contact printer of today can reach is another advantage of the technique.

Commercially available systems include TopSpot from Biofluidix (Freiburg, Germany; www.biofluidix.com), Marathon from ArrayJet (Roslin, UK; www.arrayjet.co.uk), SciFLEXARRAYER from Scienion (Berlin, Germany; www.scienion.com) and NanoPlotter from Gesim (Großerkmannsdorf, Germany; gesim-bioinstruments-microfluidics.com). The TopSpot with a printhead containing 24 reservoirs for different spotting solutions prints all probes in parallel and contactless to the substrate by a piezo actuated print mechanism. Several thousand dots can be printed without refill. Microarray printers from Arrayjet reach a very high throughput what concerns both, slides (up to 1000) and probes.

### 5.2. Contact Printing

Contact spotters use pins to deliver the probe to the surface by physical contact. Contact printing is the more technically simple and robust technique compared to non-contact printing. The method is very flexible with regards to both substrate type and hydrophobicity, and probe composition and viscosity. Required probe volumes are usually very low (e.g., 10 µL in a well of a source plate) and remaining probe in the source plate can be frozen and reused. Split pins carry the probe in a capillary and deposit a small amount of it onto the surface by tapping. Solid pins, on the other hand, are less delicate than the split pins, but have to revisit the source plate after probe deposition. Material consumption is minimized using solid pins.

Print heads with up to 192 pins are available (e.g., ArrayIt, Sunnyvale, CA, USA; www.arrayit.com). Stealth pins with various capillary dimensions can be employed for a wide range of probes including cells, beads and macromolecules. ArrayIt, e.g., offers pin with tip diameters from 37 µm up to 375 µm.

### 5.3. µ-Contact Printing (µCP)

Stamps with a bas-relief made of elastomer are used to transfer ink to a surface. The application areas are manifold, depending on the transferred pattern and the ink, which can be e.g., gold, solvents, polymers, self-assembled monolayers, but also biomolecules and cells. Usually stamps are made of polydimethylsiloxane (PDMS), a material that can easily be moulded from a master stamp with a resolution down to about 50 nm. It allows precise transfer of ink to a substrate due to its flexible nature. On the other hand, the deforming of PDMS does not allow transferring high aspect ratios, or patterns with low features in a wide distance. In the latter case hybrid stamps with a rigid backbone may add stability [72]. Another condition for efficient transfer is that the ink exhibits more affinity to the substrate than to the stamp [73]. For good printing results relative hydrophobicities of substrate and stamp [74], ink concentration, contact time, temperature, and humidity need to be optimized. An alternative approach to directly patterning proteins by µCP is creating hydrophilic and hydrophobic regions or regions which are resistant to protein adsorption by patterning of SAMs. While oligo(ethylene glycol) terminated alkanethiols are blocking the surface, methyl groups at the SAM’s tail promote protein adsorption [73]. µCP of proteins was employed to functionalize a two channel SPR chip [75]. PDMS sheets were equilibrated with the protein solution for half an hour, washed with PBS and water removing excess material and leaving a monolayer of protein on the stamp. The loaded stamp was dried under a stream of nitrogen and placed on the chip. Transfer of the protein was accomplished in 1s solely using the force of the PDMS stamp’s weight and interfacial adhesion. Automated microcontact printing for microarray applications was lately introduced by Gesim, Germany and by Biosoft Technolgies (Toulouse, France; biosoftlab.com). The InnoStamp 40 from Biosoft Technologies uses magnetic stamps which are inked, dried, aligned and finally printed with the help of a strong magnet under the printing area [76].

### 5.4. Nanobiolithography

Several groups have developed methods for the printing of nanoarrays, all of them involving atomic force microscopy (AFM). Taha et al [77] describe the writing of proteins onto aldehyde coated glass slides using a nano fountain pen (NFP), a cantilevered nanopipette controlled by an NSOM- SPM system. With the NFP it is possible to print dots and lines of biomolecules, but also etching of protein surfaces by patterning an enzyme was demonstrated [78]. Mirkin’s group at Northwestern University invented the so-called Dip-Pen Nanolithography (DPN), where an AFM probe is delivering an “ink” to a substrate. Patterning of proteins was accomplished by adsorption to DPN fabricated MHA (16-mercaptohexadecanoic acid) dots or grids [79]. Direct writing of his-tagged proteins has been achieved on nickel oxide surfaces with reasonable diffusion time [15].

Ellmark et al. [80] and Petersson et al. [81] reported on the printing of antibodies into attovials, small (diameter = 500 nm–4 µm) containers made with e-beam lithography, using nanoscale dispensing (NADIS). NADIS also uses an atomic force microscope probe with hollow cantilever and tip which deposits the probe to the surface upon contact, spots produced are in the order of 1 µm.

## 6. Sample Preparation

For many applications in protein microarray technology sample preparation can be avoided or reduced to dilution of a sample matrix with assay buffer. We have developed sensitive biomarker assays in complex matrices such as saliva, serum, plasma, urine and cell culture supernatant, without relying on sample preparation other than dilution [82]. Dilution with assay buffer is done for various purposes. First it stabilizes proteins; pH and ionic strength are adjusted to optimal conditions for the (bio-)activity of assay reagents. Second it dilutes interfering substances in the matrix. And thirdly, assay reagents such as antibodies or labelled target molecules for competitive or binding inhibition formats can be introduced. On the other hand, also low abundant analytes are diluted. Hence, an optimal dilution factor has to be found. We have been working with sample concentrations of 10% to 90% and were able to detect for instance cytokines in the pg/mL range.

Where analytes are present in the matrix at a too low concentration several enrichment procedures have been proposed, e.g., desalting; size exclusion; ion exchange [83]; filter enrichment as for instance ultrafiltration and monolithic filtration [84]; magnetic particles; and MIPs.

## 7. On-Chip Immunoassays

### 7.1. Platforms: Slides, Micro- or Nano Well Chips/Plates

**Figure 5:**
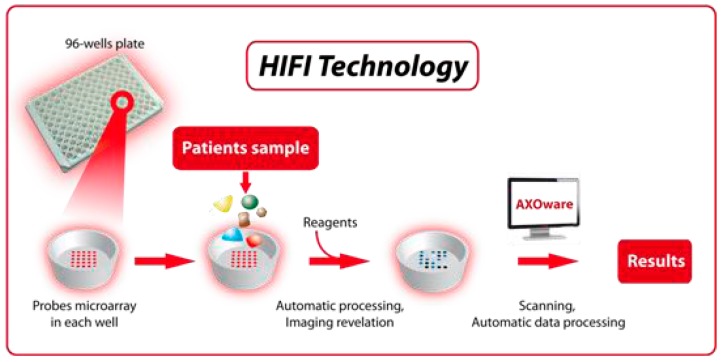
Microarray in a 96-well plate format from AXOScience: www.axoscience.com.

The choice of platforms for the immobilization of biorecognition elements is wide, not only what concerns materials (plastic, glass, metal) but also two- or three- dimensional forms and sizes: from glass microarray slides (75 mm × 25 mm) to plastic chips [85], micro- or nanotiter plates (see Figure 5) [86]; membranes; tubes (www.alere-technologies.com) [87]; microchannels [88] and more.

**Figure 6:**
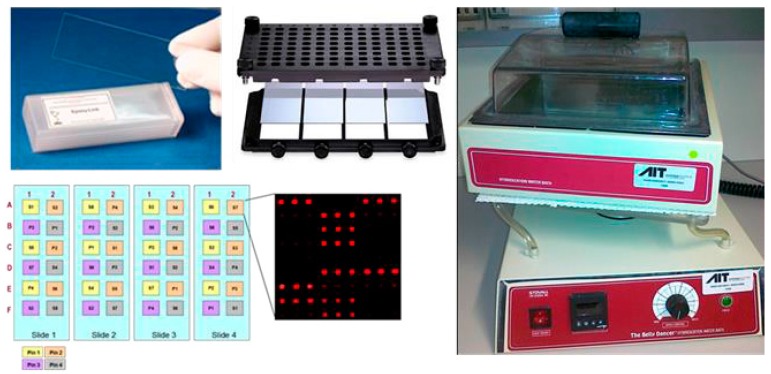
Protein microarray in a standard glass slide format, using hybridization frames (ArrayIt, Sunnyvale, CA, USA) to create up to 14 single incubation wells per slide. A four-slide set can be used for a 9-point standard curve and nine patient samples in one experiment. On the right hand side an orbital shaker with temperature controlled water bath is shown (IBI Scientific, Kapp Court Peosta, IA, USA).

Two dimensional platforms need a gasket or frame to form the reaction chambers or channels for calibration standards and samples (see Figure 6. for an example). Other than a wide range of commercial products such as EMS SecureSeal™, Corning^®^ hybridization chambers, ArrayIt^®^ hybridization frames or FastFrames™ from Whatman, self-made PDMS frames may be a cheap and flexible alternative.

In systems without microfluidics, implementation of shaking or stirring can improve assay performance considerably. An orbital shaker for this purpose, equipped with a water bath for heating, is shown in Figure 6. Stirring with magnetic particles for instance was improving assay sensitivity by a factor two in binding inhibition assays and a factor 4 in sandwich assays [89].

### 7.2. Assay Formats

In contrast to homogenous immunoassays, with all assay components being in the liquid phase, on-chip immunoassays are heterogeneous, meaning that one component is immobilized and a separation (washing) step is usually necessary. The washing step can be omitted only if the detection scheme is able to distinguish surface bound molecules or labels from molecules/labels in solution as it is the case in TIRF and confocal systems.

**Figure 7:**
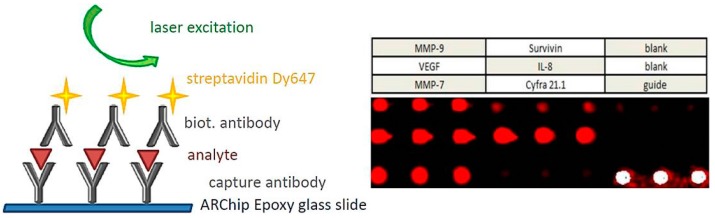
Protein chip for the detection of six biomarkers involved in bladder cancer diagnosis. Sketch of the sandwich immunoassay, array scheme, and typical fluorescence image of one array obtained after slide processing. Reproduced from [83] with permission from The Royal Society of Chemistry.

Forward phase protein arrays describe formats where a target molecule is captured by an immobilized biorecognition element and comprise direct assays (the captured biomolecule is labelled) and sandwich assays (the binding of the captured molecule is detected by a second labelled affinity reagent, see Figure 7). The signal rises with increasing abundance of the target molecules.

Binding inhibition and competitive assays are applied when only one antibody is available or the analyte is too small for providing two epitopes. In binding inhibition assays, the analyte is immobilized and competes with targeted molecules in the sample for binding of the labelled antibody. Competitive formats use labelled target molecules competing with the analyte in the sample for binding to immobilized antibodies. For both formats, the signal decreases with increasing analyte concentrations.

Reversed phase assays rely on immobilization of the target molecules and subsequent binding of a labelled antibody. Spotting of the samples (e.g., cell lysate) is followed by incubation with (labelled) specific antibodies. Here, only one Ab species per label can be detected. Reversed phase assays are mainly applied in drug discovery or screening for molecular markers.

### 7.3. Immuno-PCR

In contrast to DNA targets, to date proteins cannot be amplified directly. Too low sensitivity poses a serious problem for protein microarrays, since often very low concentrations (e.g., pg/mL; fMol/mL) have to be detected in complex matrices. When proteins involved in assays are labelled with oligonucleotides (e.g., a detection antibody is labelled with a specific DNA), those can be amplified via PCR and the DNA produced can be detected. Sano et al. showed the great potential of Immuno-PCR for high sensitivity detection of antigens already in 1992 [90]. Schweitzer and co-workers describe an adaptation of a rolling circle amplification for sensitive detection of IgE, with a limit of detection of 0.1 ng/mL, which is two orders of magnitude lower than in a conventional Enzyme-linked Immunosorbent Assay (ELISA) [91].

## 8. Signal Transduction and Read-Out

The binding event of a ligand to an immobilized biorecognition element needs to be converted into a readable signal by a transducer. Optical signal transduction is clearly dominating microarray applications, as it is a sensitive method, apt for multiplexing and not troubled by electromagnetic noise [92]. Optical detection schemes of multi-analyte affinity-based systems range from fluorescence excited by lasers or LEDs, total internal fluorescence reflectance (TIRF) [27,93], Förster resonance energy transfer (FRET) [94], absorbance [95], chemiluminescence [96] to label free techniques (e.g., interferometry, resonant mirrors and surface plasmon resonance) [97].

In chemiluminescence light is produced by a chemical reaction. The detection probe is labelled with e.g., horse radish peroxidase (HRP), upon addition of the substrate an excited state product is generated locally, which decays to a lower energy state by emitting light [98]. The advantage of chemiluminescence, a technique which has been typically used in western blotting and ELISA, is that there is no need for an expensive excitation light source or additional optics [29]. Enzymes generating colored products, often HRP or alkaline phosphatase, are conjugated to detection antibodies in colorimetric assays. Colorimetric results can be viewed by eye, but for quantification a device is needed. Portable readers, office scanners, (video) cameras, and even smartphones have been used for imaging. For the latter two changing ambient light conditions have to be compensated, for instance by conversion of RGB values into the International Commission of Illumination (CIE) 1931 color space terms [99].

Bio-layer interferometry uses the interaction of two light waves, namely the interference pattern of the light reflected from the optical layer and the one reflected from the bio-layer. Upon binding of analytes to the biolayer, this interference pattern changes in a concentration dependent way (www.fortebio.com).

With SPR technology biomolecular interactions can be observed in real-time. The technology has been commercialized by several companies (www.biacore.com, Uppsala, Sweden; www.reichertspr.com, Buffalo, NY, USA; www.bio-rad.com, Hercules, CA, USA; www.biosensingusa.com, Tempe, AZ, USA). Label free techniques such as surface plasmon resonance (SPR), and interferometry often suffer from too low detection limits and unspecific binding [96]. Fluorescent labels on the other hand may increase unspecific background, and the labelling process may alter protein function and adds to total test costs.

The labelling of antibodies itself is usually done with the active ester method. In contrast to site specific labelling of antibodies targeting the FC portion or the carbohydrate moieties, amine reactive dyes (*N*-hydroxysuccinimidester, sulfo-NHS) are attached covalently to the antibody. Purification is accomplished on a size exclusion spin column optimized for ≥40 kDa proteins.

Commercial microarray scanners usually work with helium-neon and argon lasers or use diode lasers for excitation at λ = 635 nm, λ = 532 nm and λ = 488 nm, a stage for *x-y* movements and a photomultiplier tube as detector [100]. Signals in arrays are detected pixel by pixel and pixel size may be chosen between 1 and 20 µm.

## 9. Signal Enhancement

High sensitivity is one of the most important factors of success for competitive protein microarrays in clinical applications. Strategies for signal enhancement include

- High density and accessibility of probes (i.e., immobilized biorecognition elements)
- High density of labels per binding event
- Enhanced intensity per fluorophore

3D immobilization matrices provide higher binding capacities compared to 2D surfaces but often suffer from high intrinsic background (e.g., nitrocellulose), higher unspecific binding, reduced stability and reproducibility [32]. As an alternative to hydrogels and membranes, polymer brushes with functional groups on their side chains have been developed. They can be prepared in a highly controlled way by surface initiated polymerization [45]. In Liu et al. [46] both, probe immobilization and reporter immobilization were accomplished using polymer brushes which results in high density of probes and high density of labels. A glycidyl methacrylate poly(ethylene glycol) methacrylate (GMA-co-PEGMA) copolymer was synthesized on PMMA for antibody immobilization, combining the antifouling properties of PEGMA and covalent antibody binding via the epoxy groups of GMA. The same GMA-co-PEGMA brushes were synthesized on silica nanoparticles for detection antibody binding. The synergistic amplification strategy yielded enhanced sensitivity in a sandwich immunoassay for carcinoembryonic antigen by two orders of magnitude.

The integration of nanomaterials is a promising field of research in biosensor and biochip technologies. Nanomaterials may serve as carrier or immobilization matrix for the biorecognition element [17,79], as labels [101] or energy donors [102], and often they are closely related to signal transduction and signal enhancement. Polymer nanocomposites can be functionalized with a high number of labels (as for example gold nanoparticles (AuNP), quantum dots [103,104], and organic dyes) for signal amplification.

Another approach is the development of novel biochips that exploit plasmon-enhanced fluorescence. The plasmonic structures can for instance be implemented by using cost-effective nanoimprint technology (NIL) and the resulting chip is compatible with established microarray-based fluorescence methods.

The fluorescent labels are probed by the confined field of surface plasmons that originate from collective oscillations of charge density at a surface of metallic films or metallic nanoparticles. The excitation of surface plasmons is accompanied with strongly increased intensity of the electromagnetic field which couples with fluorophores [105]. Through plasmon-enhanced fluorescence, the sensitivity of currently used assays can be enhanced by combining three effects: (a) increasing the excitation rate and decreasing background by the strongly enhanced and localized surface plasmon field intensity; (b) improving photo-stability owing to the shorter decay time of the fluorophore and (c) enhancing the efficiency of fluorescence light collecting via surface plasmon-coupled emission [106].

## 10. Miniaturization

Standard microarray formats of 25 mm × 75 mm usually harbour spots of about 50–100 µm in diameter with a spot to spot distance of 300 to 500 µm. There has been a lot of progress regarding size reduction of microarrays lately. The most important implications are

- Higher spot density and consequently higher number of BREs on a given chip size;
- Faster reaction kinetics and lower assay times;
- Reduced consumption of reagents and most important of (patient) samples;
- Small arrays avoid scanning and hence reduce size and costs of read-out instruments;
- Reduced chip size is needed for integration into (portable) instruments.

For analytical protein chips the number of probes is often low and spot/array size may not be a crucial factor when working with standard microarray scanners in central microarray facilities. For global proteome analysis, however, more than 10,000 analytes may be targeted asking for high density arrays [107]. Zhu et al. [108] for instance printed 13,000 protein samples in duplicates onto the area of a standard microscope slide for a yeast proteome microarray in order to test for protein-protein and protein-lipid interactions. Chip size reduction on the other hand is crucial firstly for reducing sample consumption and secondly for making instrumentation portable and hence independent from big laboratories. In our group we developed a test for 9 biomarkers of neonatal sepsis working with only 4 µL patient sample. Streptavidin magnetic particles allow detection of binding of biotinylated antibodies and at the same time serve as micro-stirring components [89].

In order to accommodate the more than 20,000 probes necessary for global proteome analysis, attovial antibody arrays have been developed in the group of Borrebaeck at Lund University [80]. The attovials were made by structuring 200 nm polymethyl-methacrylate (PMMA) layers on glass slides with electron beam lithography. Probes were deposited using the nanoscale dispenser NADIS (see Section 5.4 for a description of the technology). The authors discuss the limits of miniaturization, such as the number of proteins that can be captured in one spot and the maximum resolution of optical imaging. Comparing vials of 0.5 µm up to 4 µm, they achieved highest sensitivity and dynamic range with the bigger vials. Tsarfati-BarAd et al. [12] however point out the role of the immobilization chemistry, the binding site density, its homogeneity, and intrinsic non-binding area dimensions of a particular surface. As the intensity of the signal resulting from a spot is proportional to the binding area only, not to the spot area, the diameter of the spot has to be large compared to the non-binding area.

For the detection of Human Immunodeficiency Virus Type 1 p24 antigen (HIV-1 p24) in plasma Lee et al. [79] fabricated nanoarrays on gold using DPN (see 5.4 for a description of the technology). Arrays with antibodies to HIV-1 p24 antigen were produced by patterning MHA in 60 nm dots, passivating the areas around the nanodots with 11-mercaptoundecyl-tri(ethylene glycol), adsorbing the antibodies to the deprotonated MHA. The sandwich immunoassay with antigen and gold nanoparticles functionalized with polyclonal p24 antibodies was evaluated by AFM height images reaching far better sensitivity in only 1 µL of patient sample compared to a conventional ELISA.

## 11. Automated Platforms

Especially but not exclusively, for application in medical diagnostics, microarray systems will have to be fully automated in order to keep errors by users as small as possible, make measurement procedures safe for users and patients, and enhance reproducibility of read outs. Portable systems are preferred, since they enable bed-side measurements, application in outpatient care, and in doctor’s offices. Point-of-Care testing (POC), i.e., diagnostic tests at or near the patient [11], represents an especially promising field for (arrayed) biosensors. POC features several advantages such as rapid real-time analysis, no transport of patient samples, no danger of confusing samples, and no sample preparation is needed. POC testing may improve the mutual trust of clinician and patient and reduce time for therapeutic decisions [11]. To date, full integration of assays and read-out was not achieved all that often. Many systems use either lab-on-chip, integrating reagent handling and assay steps but conventional read-out with big instruments, or rely on conventional microarray slides and assays combined with portable read-out instruments.

Integration of microfluidics results in a number of advantages, such as automation of the sample processing steps, integration of mixing, reduction of assay times, and integration of read-out instruments. Microfluidic devices are fabricated from silicon, glass, or polymers. Production of plastic devices is cheaper and less time consuming, while the advantages of glass and silicon are well defined surfaces, chemical resistance, thermal stability, and excellent optical properties [109]. In contrast to static incubation, using a flow system can significantly improve assay times and detection limits, as slow diffusional kinetics hinder efficient analyte binding. Gehring et al. [110] pointed out that sensitivity improvement of at least two logs for the detection of bacteria could be achieved. Cells flowing over the capture antibodies were more efficiently recognized than in a static system. Bacterial cells feature essentially the same density as water and therefore efficiency of capturing cells at planar surfaces is very poor [110,111].

The Array Biosensor developed at the Naval Research Laboratory in Washington represents a semi-automated device. It consists of a microscope slide holding the array of BREs, a PDMS flow cell, which is addressed by a syringe needle connecting to a fluid reservoir from where the assay solutions are pumped through the flow cells. After the assay, the PDMS flow cell is removed and the microarray imaged with a CCD camera for fluorescence read-out [27,112,113]. One of the first automated platforms was the parallel affinity sensor array (PASA) with chemiluminescence detection [96]. The instrument consists of auto sampler, flow cell onto which the chip is mounted, CCD detector and computers for control and data evaluation. The prototype was used for detection of triazine herbicides contaminating water. Later an improved PASA system was used for automated analysis of 10 antibiotics in milk, achieving results in less than 5 min [114]. Determination of antibiotics in milk was demonstrated with the Munich Chip Reader (MCR), a follow up of PASA [29]. The Gauglitz group at the University of Tübingen has published numerous papers on fully automated biosensor arrays for water analysis using the optical immunosensor River Analyzer (RIANA) and the AWACSS system [26,93]. An automated electrical read-out system combined with a standard microarray on a glass slide and microfluidics has been presented by Díaz-González et al. [115]. An array of interdigitated electrode transducers measures the increase of conductivity produced by an enzyme label in each spot of the microarray.

**Figure 8:**
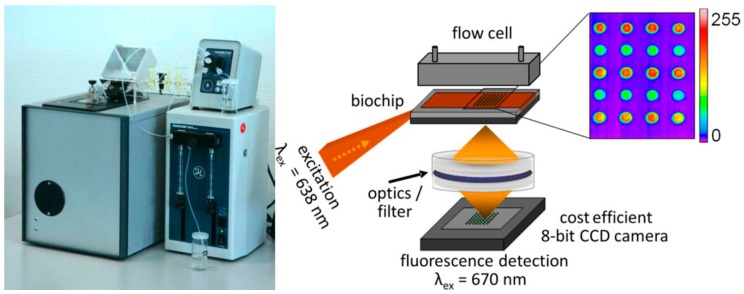
Point-of-care system for the diagnosis of sepsis. Dimensions of the instrument 38 cm × 32 cm × 40 cm. Read out: The fluorescence is excited at 638 nm by a laser coupled into a special planar waveguide biochip exciting the fluorescence by Total Internal Reflection Fluorescence (TIRF). The microarray is detected by a CCD camera via tandem objective filtering the fluorescent light. Reproduced from References [23,116] with permission from Elsevier.

With a POC device for the diagnosis of sepsis, incorporating a Total Internal Reflection Fluorescence (TIRF) detection system and a fluidic unit, the parallel detection of C-reactive protein, Interleukin-6, procalcitonin and neopterin was achieved in only 10–75 µL human plasma or serum within 25 min (instrument and scheme see Figure 8) [23,116]. In the handheld reader portMD-113 a planar waveguide is bearing the spots which are excited by the evanescent field of the guided light. Fluorescence signals are detected by portable low-cost device consisting of a pinhole array, a microlens array, an interference filter and a detector array [117].

## 12. Conclusions

During many years of research on protein microarrays for diagnostics a number of challenges emerged. Herein we identified some of them and focused the discussion on those we found pressing, namely improved reproducibility and sensitivity, antibody quality, analysis time, and integration of assays into portable systems. Other crucial topics include production cost, availability of biological or artificial biorecognition elements, crossreactivities, lack of standards, and alternative detection schemes allowing broader application of the technique. One of the recent challenges is to bring microarrays from big central research facilities to the clinical routine lab or point-of-care, which can be only accomplished by a highly interdisciplinary research bringing together new materials, nanomaterials, production techniques, and up-to date read-out and microfluidics for truly integrated diagnostic systems.

A real world example shall demonstrate in the following some issues solved as well as lingering problems. A predictive urinary biomarker panel, which can guide the physician regarding therapy and surveillance strategy of non-muscle invasive bladder cancer, has been developed. In a systems biology approach Omics data, literature data, clinical and patient data were integrated and a set of molecular processes identified, characterizing the molecular features of this disease [118]. Next, for each of this cancer associated processes biomarkers representing them were selected. Multiplexed assays for the panel were established in urine on a conventional microarray platform with fluorescence detection [83], and then transferred to a microtiter plate format enabling the easy automation of the developed multiplexed high-throughput assays and cost efficient colorimetric detection (see Figure 5) [119]. From an initial panel of 23 potential biomarkers, for seven no reagents in sufficient high quality were found, and those potential candidates had to be removed from the panel. For another two biomarkers transferring assays from buffer to the real matrix urine, resulted in decreased sensitivity and neither dilution with assay buffer nor sample preparation in order to reduce interferences of the matrix improved the performance. For one protein marker, Engrailed-2, the problem could be solved using an aptamer instead of the capture antibody, resulting in a six times lower LOD. Trying to replace the capture antibody for vascular endothelial growth factor, a very expensive reagent, with an aptamer from cost-efficient production failed however. First small scale clinical studies indicated the potential of a biomarker panel of five proteins together with three clinical parameters for discriminating recurrent and non-recurrent bladder cancer, whereas no single parameter had such ability. Yet, another shortcoming of the diagnostic biomarker panel will have to be tackled before next clinical validation steps can be initiated, namely substituting the remaining three polyclonal detection antibodies with reagents of constant performance and availability in order to ensure reproducible results.

Developed in the 1990s, protein microarrays are now on the verge of becoming a routinely used tool for a wide range of applications be it in basic or applied research. However, a number of problems remain unsolved, such as quality issues with antibody reagents, and sensitivity and reproducibility issues in real-world samples.
